# Pervaporative Dehydration of Methanol Using PVA/Nanoclay Mixed Matrix Membranes: Experiments and Modeling

**DOI:** 10.3390/membranes10120435

**Published:** 2020-12-17

**Authors:** Asmaa Selim, András Jozsef Toth, Daniel Fozer, Agnes Szanyi, Péter Mizsey

**Affiliations:** 1 Environmental and Process Engineering Research Group, Department of Chemical and Environmental Process Engineering, Faculty of Chemical Technology and Biotechnology, Budapest University of Technology and Economics, H-1521 Budapest, Hungary; andrasjozseftoth@edu.bme.hu (A.J.T.); daniel.fozer@edu.bme.hu (D.F.); agnes.szanyi@mail.bme.hu (A.S.); mizsey@edu.bme.hu (P.M.); 2 Chemical Engineering Department, National Research Centre, 33 El Buhouth Street, Cairo 12622, Egypt; 3 Institute of Chemistry, University of Miskolc, H-3513 Miskolc, Hungary

**Keywords:** mixed matrix membranes, laponite nano-silicate clay, methanol dehydration, solution–diffusion model, pervaporation mathematical modeling

## Abstract

Encouraged by the industrial problem of removing water from methanol solutions, a simple exfoliation method is applied to prepare polyvinyl alcohol (PVA)/laponite nanoclay mixed matrix membranes (MMMs). The membranes are used for the pervaporative dehydration of the methanol-water solution. The influence of the nanoclay content on the pervaporation performance is investigated. The results show that the PVA10 membrane containing 10 wt% Laponite loading exhibits excellent separation efficiency; therefore, all the experimental work is continued using the same membrane. Additionally, the effects of feed concentration and temperature on methanol dehydration performance are thoroughly investigated. The temperatures are ranging from 40–70 °C and the water feed concentrations from 1–15 wt% water. A maximum separation factor of 1120 can be observed at 40 °C and the feed water concentration of 1 wt%. Remarkably, two solution–diffusion models, the Rautenbach (Model I) and modified method by Valentínyi et al. (Model II), are used and compared to evaluate and describe the pervaporation performance of the mixed matrix membrane. Model II proves to be more appropriate for the modeling of pervaporative dehydration of methanol than Model I. This work demonstrates that PVA/nanoclay mixed matrix membranes prepared can efficiently remove water from methanol aqueous solution with pervaporation and the whole process can be accurately modeled with Model II.

## 1. Introduction

Recently, the treatment of ethyl-acetate–methanol-water mixture starts to be a severe problem in the pharmaceutical industry. The non-ideal mixture is produced in significant amounts, which means an actual environmental issue for the industry sector. The first step, ethyl-acetate–water separation, is already described [1]. The heterogeneous binary azeotrope can be enriched in the overhead product without methanol with extractive heterogeneous-azeotropic distillation technique [2] while separating the methanol/water solution, which is the bottom fraction is still a problem. However, methanol and water do not form an azeotrope. They can be separated by conventional distillation, but the distillation separation is cost demanding because of the low relative volatilities of methanol and water. 

Pervaporation is vastly accounted for liquid separation in different ways, such as the dehydration of the chemical solvents or the concentration of volatile organic compounds, VOCs, or the separation of two organic solvents using hydrophilic, hydrophobic, and organoselective membrane, respectively [3,4,5]. 

Pervaporation (PV) has been reported as a portentous process for alcohol separation and dehydration [6,7,8]. Pervaporation is vital to improve the alcohol composition from 80–85 wt% to 99 wt%. However, the traditional distillation is working efficiently up to the same concentration and above. The process becomes prohibitively expensive, especially near an azeotropic composition. Therefore, a significant share of the production cost goes to alcohol purification [9,10].

Additionally, based on literature, pervaporation operating cost could be 50% less than the conventional distillation with relatively higher separation efficiency [11]. Further, PV has attained much attentiveness due to its preference for lower energy consumption and higher efficiency and its merit as eco-friendly and economically undisputed [12,13]. Particularly ethanol and isopropanol are the most studied alcohols for pervaporation dehydration. While for methanol, only a few studies were reported. As a result that methanol (MeOH) has a relatively similar polarity to water, molecular weight results in competing for water in the adsorption step to the membrane surface. The separation principal of pervaporation is based on the difference in the polarity of the compounds which need separation, their molecular size, and the affinity of the most polar substances for the interface of the membrane. As far as the polarity is concerned, methanol is the water’s closet neighbor. Based on the experience gained, Sulzer Chemtech has developed a new generation membranes which can dehydrate methanol from organic substances [14].

In the PV process, the driving force is generated on the membrane sides; mainly, the feed mixture is heated up to a specific temperature then penetrates through the membrane to be converted to gas and leave from the permeate side. By maintaining the vacuum, the collected vapors are condensed by a cold medium [15]. Hence, the membrane is the core of the PV process; its type, material, and intrinsic properties are the most critical factors to achieve high separation performance.

For the dehydration process, usually, hydrophilic polymers are used, mostly polymers with high affinity towards the water such as poly (vinyl alcohol) (PVA), chitosan, and alginate, which increase the PV performance. However, these materials reported having low mechanical stability in aqueous solutions [16]. This is credited to their high sorption affinity towards the water and low rigidity of those polymers, which results in lowering the water selectivity and increase the permeability in a trade-off trend [17]. In an attempt to inhibit this phenomenon, different modifications are reported, such as chemical cross-linking, heat treatment, blending with another polymer, grafting [18,19,20], as well as mixing with inorganic fillers [15,21]. 

Mixed matrix membranes consisting of polymer matrix filled with inorganic fillers were firstly reported by Kulprathipanja et al. to combine the advantages of both polymeric and inorganic membranes [22]. The most-reported polymer for PV dehydration technology is the well-studied hydrophilic PVA, followed by other polymeric materials such as chitosan and alginate [23]. Among the variety of inorganic fillers, nano-silicate clay types such as clinoptilolite, montmorillonite, and bentonite are perceived. Using the nanoclay as fillers for PVA is a good filler for the dehydration process due to their unique characteristics, exceptionally high surface area, and biocompatibility [24,25,26].

Laponite is a relatively new nano-silicate clay, has a disc structure with 30 nm diameter and 1 nm in thickness with empirical formula Na + 0.7[(Mg5.5Li0.3) Si8 O20 (OH) 4] − 0.7. Laponite clay has a high affinity towards the water and forms a clear dispersion easily in water. Laponite is reported for enhancing the mechanical and the physical stability of polymer nanocomposite and hydrogels [27,28,29,30]. Silicate nanoclay is devilishly reported for biochemical and biological applications such as wound healing and drug delivery [31,32,33]. However, to our best of knowledge, no research has studied for methanol dehydration applications.

Due to the fact, methanol has an almost double molecular weight as well as the solubility parameter difference (Table 1), that leads to difficulty in the separation [14].

The mechanism of component separation in a liquid mixture by pervaporation is complex but consists of the following steps [16,35]: (1) sorption of the vital component in the membrane; (2) preferential diffusion of the element through the membrane material; (3)desorption and evaporation of the element on the permeate side into the vapor phase. 

For pervaporation modeling, mainly the empirical models are found, such as the pore flow model, total solvent, and volume fraction model [35,36,37,38]. The most accepted, recommended, and resemble the most the real phenomenon is the solution–diffusion model [16], which can be applied only for two-layered composite membranes.

This paper reports: (1) the emerging process of methanol pervaporation dehydration using PVA/nanoclay mixed matrix membranes with different clay content and under different operating temperatures ranging from 40–70 °C, (2) provides sufficient understanding of the modeling of methanol dehydration with pervaporation using mixed matrix membranes following the three main steps of modeling identification from laboratory experiments, parameter estimation using ChemCAD and Statistica programs, and verification by comparing the modeled and measured data.

## 2. Experimental

### 2.1. Material

Poly (vinyl alcohol) (85,000–124,000 g/mol, 99% + hydrolyzed) is purchased from Sigma-Aldrich Chemie GmbH. (Schnelldorf, Germany). Laponite XLG (ρ = 2.53 g/cm^3^, CEC = 0.55 meq/g, d = 25–30 nm, h = 0.92 nm) is kindly supplied by the Laboratory of Plastics and Rubber Technology, Department of Physical Chemistry and Materials Science, Budapest University of Technology and Economics. Methanol (MeOH) absolute is provided by VWR Chemicals (Budapest, Hungary). All chemicals are used as such without further purification.

### 2.2. Membrane Fabrication

Following the solution-casting procedure, both the plain PVA and the mixed matrix membranes with different wt% of laponite clay with respect to PVA via phase inversion were fabricated. Briefly, the polymer powder was dissolved in deionized water to prepare a 5% wt PVA solution. For the mixed matrix membranes, the prepared PVA solution was mixed with clay solution with different Laponite content (2, 5, 7, and 10 wt% with respect to the dry PVA weight) after being sonicated for 3 h. The mixed solution was stirred for 24 h and cast to obtain membranes. All of the membranes are dried at room temperature for 48 h. The final dry membranes are picked up and designated as PVA0, PVA2, PVA5, PVA7, and PVA10, corresponding to the laponite content in the membrane. For better performance, the only pristine membrane is thermally cross-linked at 60 °C for 3 h, and the membrane is designed as PVA. 

### 2.3. Swelling Measurements

Swelling measurements were carried out by completely drying the membrane at room temperature and weighed and then immersed in methanol solutions of 1, 5, 10, and 15 w% water in a sealed vessel at room temperature. After 0.5, 1, 2, 3, 4, 5, 6, and 8 h, the membranes were taken out and dried carefully with tissue paper to remove the surface solution and weighted as fast as possible, and immersed in the mixture solutions again. Each membrane was measured three times, and the average was taken as the final result. The degree of swelling percentage is calculated by the following equation:

(1) $$DS\%=\;\frac{M_{s}-M_{d}}{M_{d}}*100$$

where *M_s_* and *M_d_* are the mass of the swollen membrane and the dry one, respectively. 

### 2.4. Pervaporation Tests

**Figure 1:**
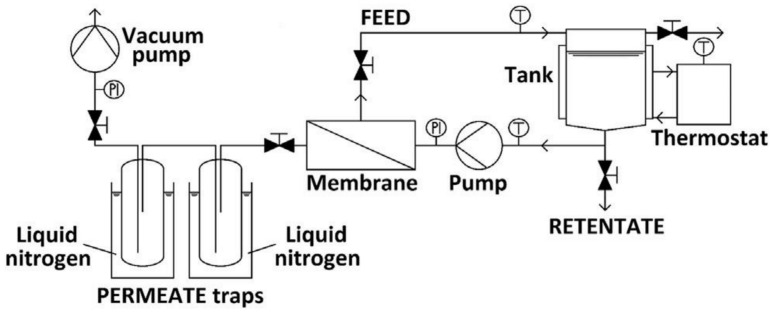
Schematic model of CM-Celfa P-28 Membrantechnik AG in pervaporation mode.

All the pervaporation experiments are performed using a multifunction lab-scale P-28 apparatus supplied by CM-Celfa Membranetechnik AG, shown in Figure 1 and described elsewhere [39]. A feed of 500 mL MeOH/water mixture is loaded in a double jacketed feed tank stirred and circulated through the system diminish the concentration and temperature polarization. The permeate pressure of 0.27 kPa (2 torrs) was obtained by applying a vacuum to ensure that the required driving force is achieved across the membrane. On the permeate side, a liquid nitrogen cold trap is used to collect the condensed vapor in the liquid form. Before conducting experiments, the membrane is swollen in the feed solution for an hour.

Additionally, the circulation system is turned on until reaching the required temperature and stabilized for another hour to ensure the stable condition before sample collection. The temperature is maintained by a water thermostat and checked using a thermometer on the inlet and outlet of the apparatus. The permeate samples are analyzed when collecting enough condensate after conditioning the membrane for at least 2 h. The concentration of the feed and permeate is measured by the RA-620 (accuracy ±0.00002, KEM Kyoto Electronics, Tokyo, Japan) refractometer.

All experiments are repeated three times to ensure reproducibility. To evaluate the pervaporation performance, total flux (*J*) and separation factor (*β*) are employed. The flux, *J* (g/m^2^·h) depends on the permeate weight, *W* (g), effective area of the membrane, *A* (m^2^), and experiment duration, *t* (h) and is obtained using the following equation:

(2) $$J=\;\frac{W}{A\times t\;}$$

The separation factor (*β*) is calculated using the following equation:

(3) βi,j= YiYjXiXj

where $Y_{i}\;$ and $X_{i}$ are permeate and feed mass fractions of component *i*, while *i* and *j* refer to water and alcohol, respectively. The Pervaporation overall Separation Index (PSI) is expressed by

(4) $$PSI=J\left(\beta -1\right)$$

For evaluating the intrinsic properties of the MMMs, both water and alcohol permeabilities are calculated using the following equation:

(5) $$P_{i\;}=\;\frac{J_{i}\;\delta }{\left(x_{i}\gamma_{i}P_{i}^{sat}-y_{i}P^{p}\right)}$$

where $P_{i\;}$ (g/m·h·kPa) represent component *i* permeability, δ (m) is the thickness of the membrane, $J_{i}$ (g/m^2^·h) is the individual flux, $\gamma_{i}$ is the activity coefficient, $P_{i}^{sat}$ (kPa) is the saturated vapor pressure, $x_{i}$ and $y_{i}$ are the mole fraction in the feed and permeate side, respectively. $P^{p\;}$ (kPa) represents the downstream pressure. The activity coefficients are calculated using the Wilson equation while the $P_{i}^{sat}$ is calculated from the Antoine equation, using ChemCAD software. The selectivity ($\alpha_{ij}$) is calculated from the ratio between *i* and *j* permeability, where *i* and *j* are the water and alcohol, respectively.

(6) $$\alpha_{ij}=\;\frac{P_{i}}{P_{j}}$$

## 3. Modeling of Pervaporation

The methodology of Rautenbach [16] and Valentinyi et al. [40] are selected for modeling of pervaporation. Equation (7a,b) show the basic formula of these two models:

(7a) $$J_{i}=\frac{1}{1+\left\{\left[{\bar{D}}_{i}\right]/\left(p_{i0}\cdot {\bar{\gamma }}_{i}\right)\right\}}\cdot \frac{\left[{\bar{D}}_{i}\cdot \right]}{{\bar{\gamma }}_{i}}\cdot \left(\frac{p_{i1}-p_{i3}}{p_{i0}}\right)\;i\;=\;\left(1,\ldots ,k\right)$$

(7b) $$J_{i}=\frac{1}{1+\left\{\left[{\bar{D}}_{i}\cdot exp\left(B\cdot x_{i1}\right)\right]/\left(p_{i0}\cdot {\bar{\gamma }}_{i}\right)\right\}}\cdot \frac{\left[{\bar{D}}_{i}\cdot exp\left(B\cdot x_{i1}\right)\right]}{{\bar{\gamma }}_{i}}\cdot \left(\frac{p_{i1}-p_{i3}}{p_{i0}}\right)\;i=\left(1,\ldots ,k\right)$$

This PV model is the development of the Rautenbach model [16]. The improvements considering the temperature dependencies of the PV and concentration dependencies of the transport coefficient [10,32]. The basic Rautenbach model (Model I) and the improved one (Model II) are used for modeling our experiments.

Partial pressures ($p_{i0}$) are calculated according to the Antoine equation:

(8) $$p_{i0}=exp\left[A+\frac{\;B}{T}+\mathrm{Cln}T+DT^{E}\right]*10^{-5}$$

where A, B, C, D, E are material depending constants. Transport coefficient ($\bar{D_{i}}$) depends on the temperature in an Arrhenius type exponential way.

(9) $$\bar{D_{i}}=\bar{D_{i}^{*}}exp\left[\frac{E_{i}}{T}\left(\frac{1}{T^{*}}-\frac{1}{T}\right)\right]$$

In Equation (9), $T^{*}$ is the reference temperature, equal to 293 K and $E_{i}$ is the activation energy for component i and is associated with the transport coefficient. The liquid activity coefficients can be calculated with different vapor–liquid equilibrium models or with the Wilson equation. A detailed description of the semi-empirical PV model can be found in [40,41].

Activation energies, transport coefficients, and in the case of Model II for both compounds, the B parameters show the concentration dependencies of the transport coefficients, which are estimated based on our measured data. The nonlinear estimation process is used by defining a user-specified regression custom loss function (Equation (8)) in STATISTICA^®^ program environment. The model verification can be obtained with objective function (OF), which is minimized the deviation of the modeled and the measured values.

(10) $$OF=\;\overset{n}{\underset{i=1}{\sum }}\left(\frac{J_{i,measured}-J_{i,modelled}}{J_{i,measured}}\right)$$

The improved model was tested by Ashraf et al. [42] with two dehydration systems: 83–98 m/m% 1-butanol and 85–97 m/m% isobutanol over Sulzer™ PERVAP 2510 (PVA).

## 4. Results and Discussion 

### 4.1. Influence of Laponite Content on the Dehydration of Methanol Aqueous Solution

**Figure 2:**
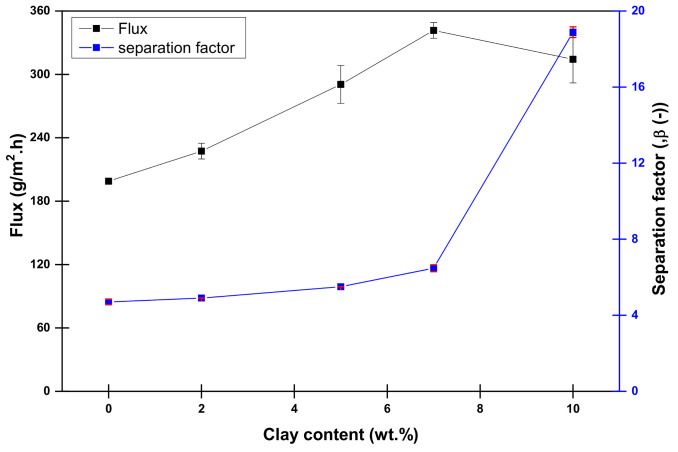
Dehydration performance of the PVA membrane and laponite mixed matrix membranes (MMMs) at 40 °C using 85 wt% MeOH as feed composition.

Hence, the pristine PVA0 membrane swells quickly; therefore, all the pervaporation tests are done using the thermally cross-linked membrane PVA as 0% clay. The performance of dehydration separation of 85 wt% methanol solutions at 40 °C as a function of the nanoclay concentration in the casting solution have been investigated and presented in Figure 2. Both the flux and the separation factor are following a similar trend. Both separation factor and permeation flux are increasing with increasing the laponite concentration in the casting solution. The increment of the flux could be attributed to the increase of the hydrophilicity of the membrane due to the incorporation of the hydrophilic laponite clay. While the increase in the separation factor is because of (1) the decrease in the free volume in the polymer matrix upon the laponite loading and (2) the lower diffusivity of methanol in the membrane than water diffusivity. However, the flux is relatively constant then slightly decreases with the further addition of laponite at 10 wt%. The reason for that could be the aggregation of the nanoclay and the formation of the nanoclay layer between the polymer matrix, as reported [43,44]. The same reason increases the separation factor significantly after increasing the laponite content as the laponite layer works as an additional filter inside the polymer matrix. Hence, PVA 10 shows the highest separation factor and good permeation flux; it will be used for all the upcoming performance evaluation.

### 4.2. Swelling of PVA 10 Mixed Matrix Membranes in Methanol/Water Mixtures

**Figure 3:**
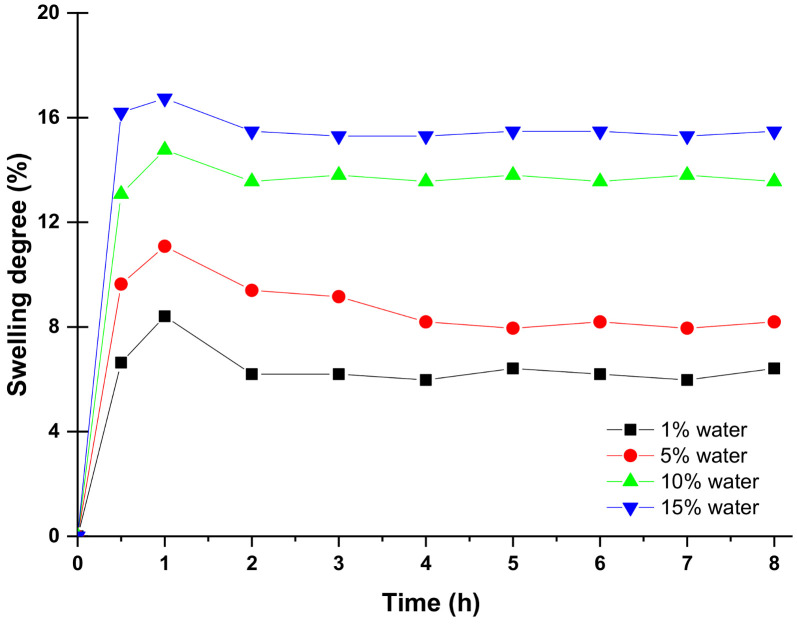
Swelling behavior of PVA10 as a function of water content in the feed.

Generally, hydrophilic membranes swell in water and polar organic solvent. Hence, the swelling has a significant impact on the pervaporation dehydration performance by affecting solubility and selectivity of the membranes towards feed components. Swelling tests are performed at room temperature to understand the affinity PVA10 towards different water/methanol concentrations and the mutual interaction. Figure 3 shows the results of the average of three parallel measurements for swelling behavior of PVA10 membrane at 1, 5, 10, and 15 wt% water/methanol solutions. It can be seen the swelling degree is increasing with increasing the water content in the feed due to the increased affinity of the membrane towards water [45,46]. Additionally, it is recognized that after 0.5–1 h, the degree of swelling is practically constant.

### 4.3. Effect of Temperature and Feed Concentration at the Pervaporation Performance

**Figure 4:**
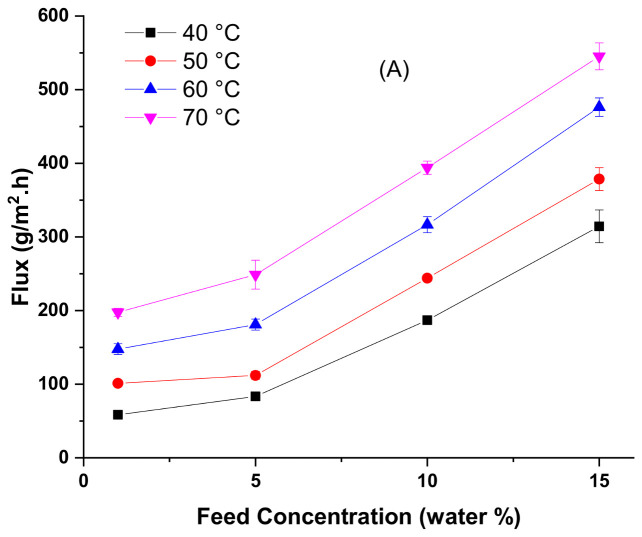

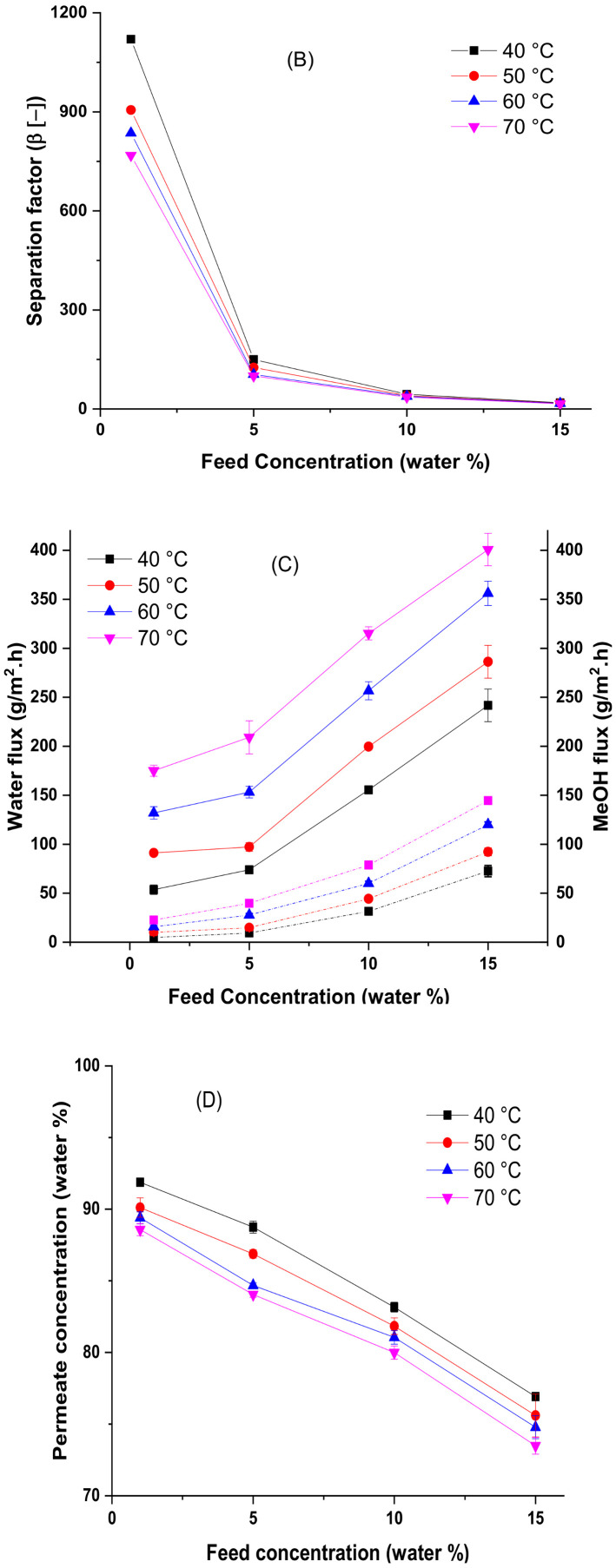

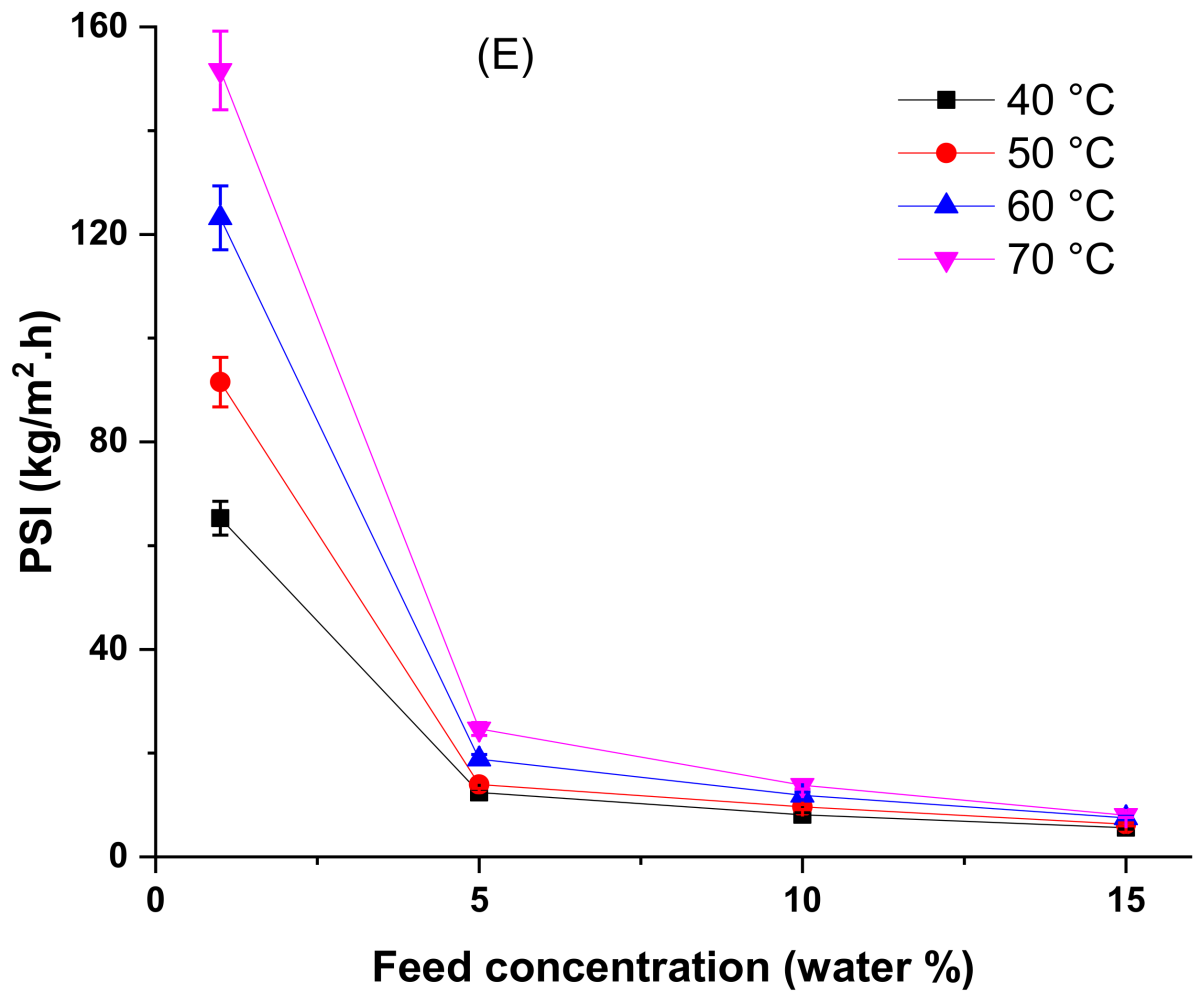
Methanol dehydration performance of PVA10 membrane at a different temperature as a function of feed water concentration vs. (**A**) total flux, (**B**) separation factor, (**C**) water and methanol fluxes (continuous line water flux; dotted line methanol flux), (**D**) water permeate concentration, and (**E**) pervaporation separation index.

Although the membrane material and characterization are the most crucial factor in the pervaporation process; nevertheless, the operating temperature could be considered as a predominant feature in the pervaporation process [38]. Hence, changing the temperature can directly affect the driving force, the permeation flux, water permeability, and diffusivity through the membrane. The influence of operating temperature and concentration of water in the feed solution on methanol dehydration performance are investigated and shown in Figure 4. Additionally, it can be seen that changing the temperature has a considerable impact on solvent and water vapor pressures, which also affects the thermodynamic properties of the feed [47].

On the other hand, it is observed from Figure 4C that the flux for both feed components is increasing with increasing of the water concentration in the feed mixture. The reason behind that behavior is the fact that the PVA10 membrane has a high affinity towards the water, so increasing water concentration will result in enhancing the membranes swelling, as shown in Figure 3. Consequently, strengthening the permeation for both water and methanol through the membranes leads to decreased separation factors. Moreover, the influence of both the temperature and the water concentration in the feed solution on the pervaporation separation indexes (PSI) for all the membranes are gathered in Figure 4E.

**Figure 5:**
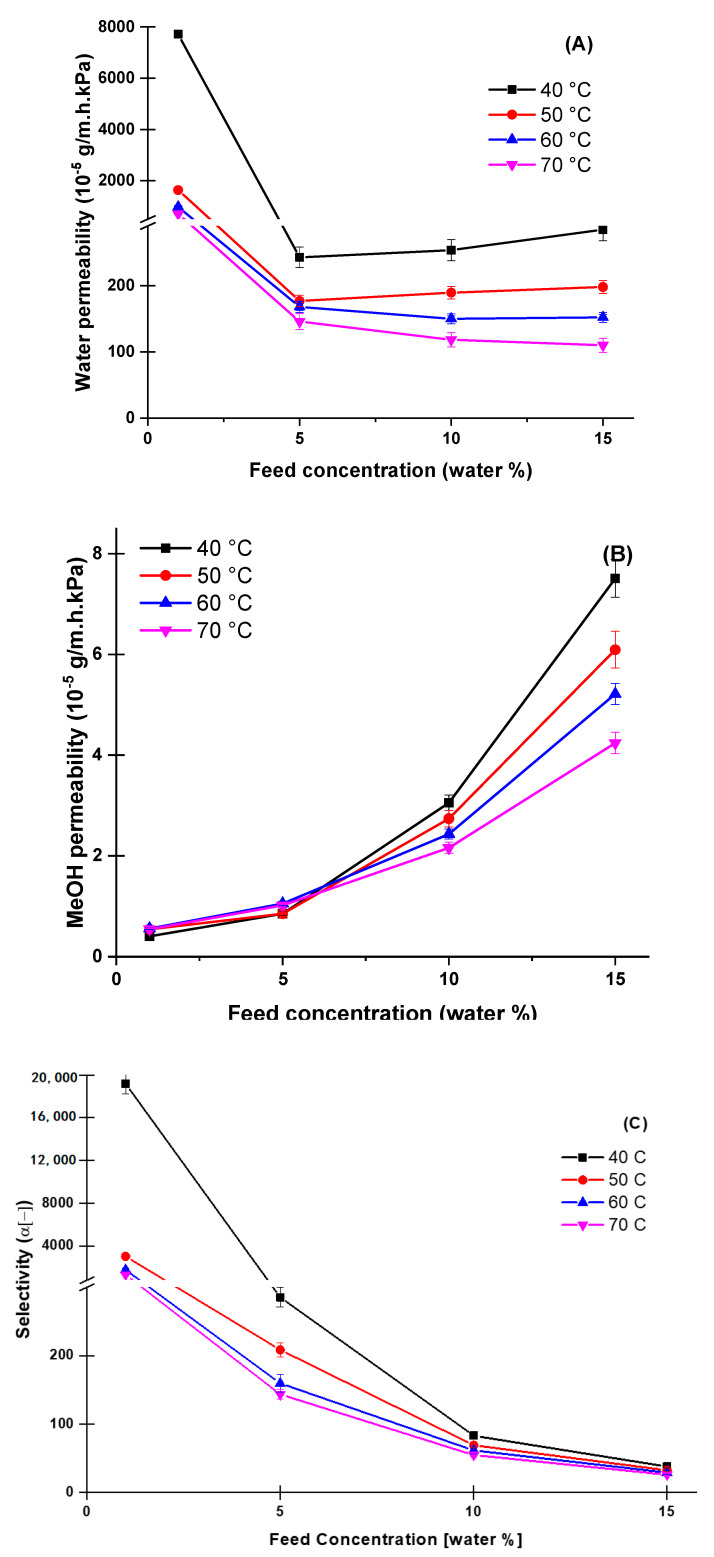
Intrinsic properties; water permeability (**A**), methanol permeability (**B**), and Selectivity(**C**) of PVA10 as a function of water concentration in the feed solution at temperature ranges from 40–70 °C.

Alternatively, the pervaporation performance based on the intrinsic properties of the PVA10 membrane is demonstrated such as permeability (P) selectivity (α) for both water and methanol as a function of operating temperature and water content in the feed, and the results are shown in Figure 5A–C. Distinctly, the selectivity is following the separation factor with increasing the feed water content. Although the methanol permeability follows the methanol flux behavior, water permeability follows a downward trend with increasing the water in the feed. Simultaneously, both water and methanol permeabilities are having a contrary action of decreasing at elevated temperatures.

### 4.4. Comparison of Pervaporation Dehydration Performance of Aqueous Methanol Solutions

Until now, using polymeric pervaporation membranes for methanol dehydration has not been widely studied because of the lack of suitable membrane material, which combines good economic availability and anti-swelling towards methanol [48,49,50]. However, in this work, we demonstrate that PVA10 shows a high flux as well as a comparable separation factor for methanol dehydration.

Table 2 shows a comparison between the results demonstrated from this work and some of the reported experimental data for the pervaporation dehydration of the methanol-water mixture. From the table, PVA10 is showing a significantly higher fluxes and is acceptable compared to PPSU and its crosslinked membranes. However, compared to the POLYIMIDE/UiO66-NH2 mixed matrix membrane, PVA10 obtains higher fluxes and separation factor. Surprisingly, comparing PVA10 to the commercial Sulzer membrane, PVA10 shows approximately 10 timer higher separation factor and same magnitude for flux.

### 4.5. Modeling of Pervaporation Using PVA/Laponite Nanoclay MMM

**Figure 6:**
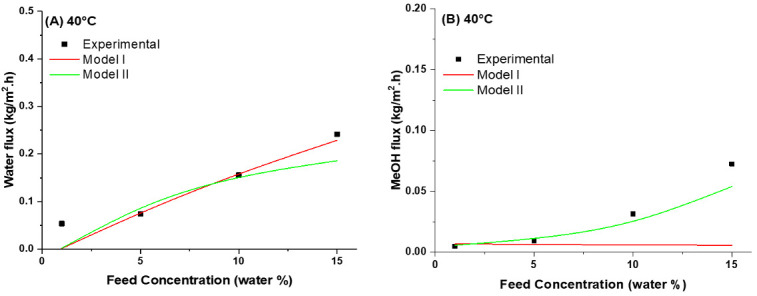

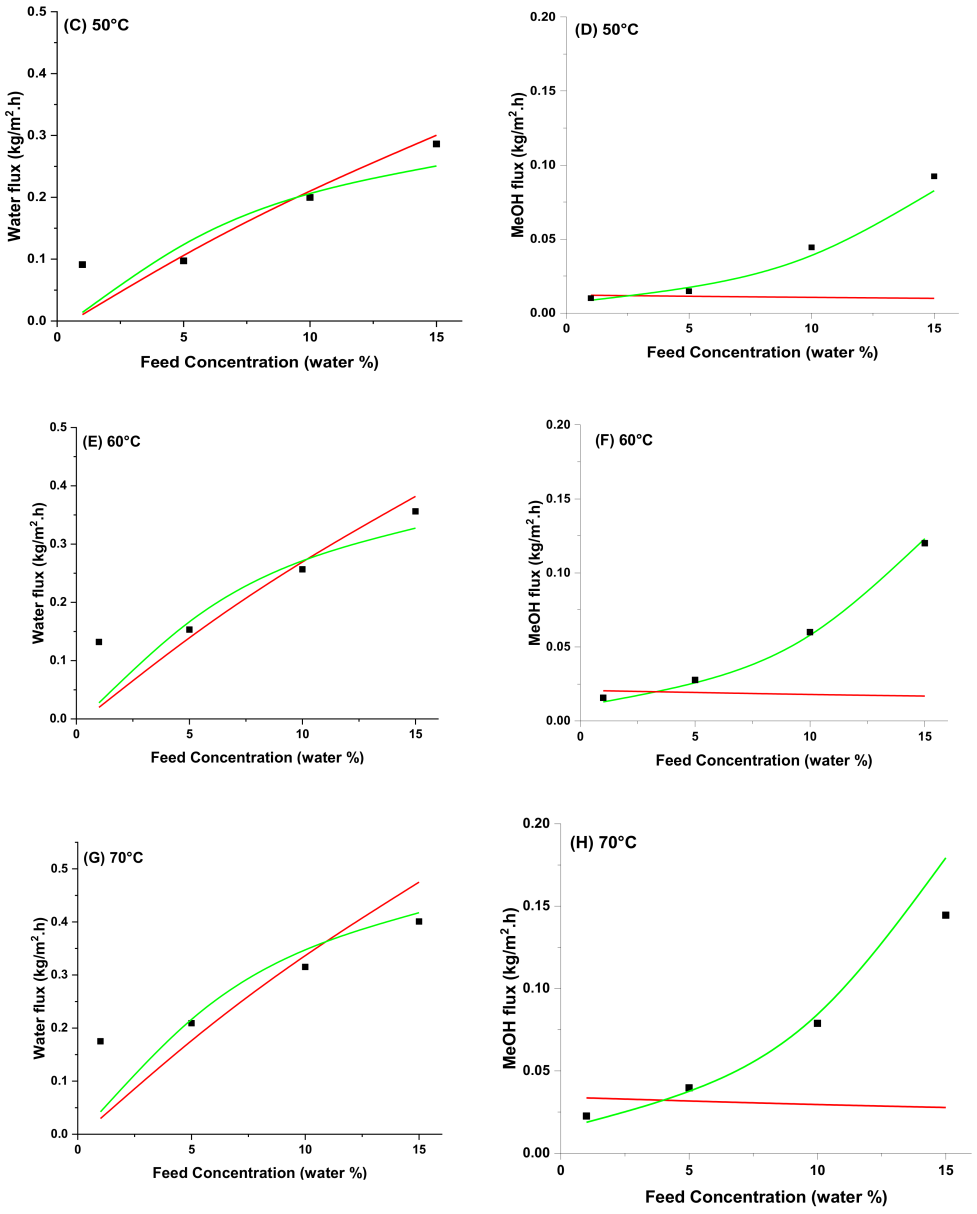
Comparison between measured partial fluxes (■) calculated from Model I (**Red**) and Model II (**Green**); (**A**,**C**,**E**,**G**) water fluxes, and (**B**,**D**,**F**,**H**) methanol fluxes as a function of feed water concentration using PVA10 MMM at operating temperature of 40−70 °C.

Both the Rautenbach model [16] and the modified model [40] were applied on the experimental results, and the estimated values of the model parameters such as transport coefficients, activation energies, and exponential parameters of the two models as well as the minimized objective functions are summarized in Table 3. The partial fluxes for both methanol and water as a function of the feed water of the experimental results are compared with the calculated results from both models at different operating temperatures. The comparison results are presented in Figure 6A–H.

The results in Figure 6 and Table 3 demonstrate that for the dehydration of methanol using mixed matrix membranes, the Rautenbach model (Model I), which using the transport coefficient as a constant, is less appropriate in describing the pervaporation. While the modified model (Model II) is much more suitable due to considering the concentration dependence of the transport coefficient for pervaporation dehydration of methanol. Hence, it is widely reported the exponential correlation between diffusion coefficient and feed concentration, so the modified model considering this is more accurate for modeling of pervaporation. It can be seen from Table 3 that the objective functions are lower for the modified model (Model II) [40,54].

## 5. Conclusions

This study is confirming the possibility to successfully improve the pervaporative dehydration of methanol using mixed matrix hydrophilic membranes. PVA/laponite nanocaly membranes are simply prepared by the exfoliation method. The efficient nanoclay content and preferential sorption of water of the PVA membranes result in a high separation factor as well as total fluxes. The mixed matrix membrane with a laponite nanoclay loading of 10 wt% shows the highest separation factor and excellent total flux. The total flux of the fabricated PVA10 membrane ranges from 0.06 to 0.55 kg/m^2^ h using a water feed concentration from 1–15 wt% at 40–70 °C.

However, a maximum separation factor of 1120 can be achieved for a feed of 1 wt% water content at 40 °C. The results show that total flux and partial fluxes are influenced by the temperature and the water concentration of the feed solution, while the separation factors and selectivity exhibit a contradictory behavior. This can be attributed to the high affinity of the membrane towards the water, so increasing water concentration enhances the swelling of the membrane. Therefore, it promotes the permeation for both water and methanol through the membranes and results in lower separation factors and selectivity.

Comparing the experimental results obtained by PVA10 to different types of membranes show that PVA10 has high flux as well as a comparable separation factor for methanol dehydration at the same operating conditions. Additionally, PVA10 shows approximately 10 times higher separation factor and same magnitude for flux compared to the commercially available Sulzer membrane. 

The pervaporation performance of PVA10 membrane can be accurately characterized by the generalized solution–diffusion model modified by Valentinyi et al. [44]. This model proves to be more capable for the pervaporation modeling using mixed matrix membrane based on the results of parameter estimation and better fitting to the experimental data. It can be concluded that the prepared PVA/nanoclay membranes enable the efficient removal of water from methanol aqueous solution using pervaporation technology.

## Figures and Tables

**Table 1:** Hansen’s solubility parameters for pure alcohols [34].

Solvent/Polymer	δd [MPa0.5]	δp [MPa0.5]	δh [MPa0.5]	δt [MPa0.5]
Methanol	15.10	12.30	22.30	29.61
Ethanol	15.80	8.80	19.40	26.52
IPA	15.80	6.10	16.40	23.58
Water	15.50	16.00	42.40	47.90

**Table 2:** Comparison of membranes for pervaporative dehydration of methanol-water mixture.

Membranes	T	F_water_	J_total_	*β*	Reference
	[°C]	[wt%]	[kg/m^2·^h]	[–]	
**5% SPPSU**	60	~15	0.03	11.1	[48]
**PPSU**	60	15	0.05	28.7	[49]
**H-PESU**	60	15	0.06	31.3	
**PAI-PEI HOLLOW FIBER**	60	15	1.03	4.71	[51]
**SODIUM ALGINATE/PVA**	60	10	0.03	135	[52]
**SULZER PERVAP-2201**	60	10	0.50	3	[53]
**POLYIMIDE/UIO66-NH2 (10%)**	60	15	0.17	13.22	[50]
**PVA10**	60	10	0.32	38.52	This study
		15	0.48	16.8	
	70	10	0.4	36	
		15	0.55	15.72	

**Table 3:** Estimated parameters for a methanol−water mixture using the PVA10 mixed matrix membrane.

Function	Model I		Model II	
	Water	MeOH	Water	MeOH
D [kmol/m^2·^h]	2.63 × 10^−2^	6.90 × 10^−5^	3.38 × 10^−2^	4.10
E_v_ [KJ/Kmol]	21,068.62	49,344.13	23,435.44	37,481.79
B [-]			−2.18	−11.06
OF-Water	3.24		3.04	
OF-MeOH	6.01		0.39	

## Nomenclature

A	Membrane transfer area $\left[m^{2}\right]$
B	Constant in Model II $\left[-\right]$
${\bar{D}}_{i}$	Transport coefficient of component i $\left[\mathrm{kmol}/\left(m^{2}\cdot h\right)\right]$
${\bar{D}}_{i}^{*}$	Relative transport coefficient of component i $\left[\mathrm{kmol}/\left(m^{2}\cdot h\right)\right]$
$E_{i}$	Activation energy of component i $\left[\mathrm{kJ}/\mathrm{mol}\right]$
*F*	Feed
i	Component number
j	Component number
$J_{total}$	Total flux $\left[g/\left(m^{2}\cdot h\right)\right]$
$J_{i}$	Partial flux $\left[g/\left(m^{2}\cdot h\right)\right]$
P	Permeate
$p_{i0}$	Pure i component vapour pressure $\left[\mathrm{bar}\right]$
$p_{i1}$	Partial pressure of component i on the liquid phase membrane side $\left[\mathrm{bar}\right]$
$p_{i3}$	Partial pressure of component i on the vapour phase membrane side $\left[\mathrm{bar}\right]$
$p_{3}$	Pressure on the permeate side $\left[\mathrm{bar}\right]$
R	Retentate
Ʀ	Gas constant $\left[\mathrm{kJ}/\left(\mathrm{kmol}\;K\right)\right]$
t	Time $\left[h\right]$
T	Temperature $\left[℃\right]$
$T^{*}$	Reference temperature: 293 K
$x_{i1}$	Concentration of component i in the feed $\left[\mathrm{wt}\%\right]$

## Abbreviations

OF	Objective function
PSI	Pervaporation Separation Index $\left[\mathrm{kg}/\left(m^{2}\cdot h\right)\right]$
PVA	Polyvinyl alcohol
PV	Pervaporation
**Greek letters**	
α	Selectivity
β	Separation factor
${\bar{\gamma }}_{i}$	Average activity coefficient of component i
$\gamma_{i1}$	Activity coefficient of component i in the feed
δ	Membrane thickness $\left[\mu m\right]$
